# Analytical Mapping of Swiss Hard Cheese to Highlight the Distribution of Volatile Compounds, Aroma, and Microbiota

**DOI:** 10.1021/acs.jafc.4c10980

**Published:** 2025-03-11

**Authors:** Lucie K. Tintrop, Marco Meola, Mireille T. Stern, Monika Haueter, Noam Shani, Hélène Berthoud, Barbara Guggenbühl Gasser, Pascal Fuchsmann

**Affiliations:** † Agroscope, Schwarzenburgstrasse 161, Bern 3003, Switzerland; ‡ DATABIOMIX, Zürcherstrasse 39D, Schlieren 8952, Zürich, Switzerland; § Swissmedic, Hallerstrasse 7, Bern 3012, Switzerland

**Keywords:** volatilome, volatile distribution, microbiota distribution, sensory analysis, hard cheese, vacuum in-tube extraction, gas chromatography−mass spectrometry

## Abstract

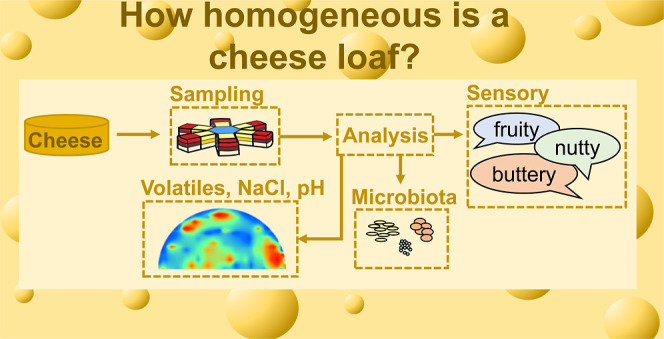

Cheese is one of the most consumed fermented animal-based products globally, rendering its quality assessment and evaluation of substantial economic interest. Understanding the degree of cheese homogeneity is paramount for designing effective sampling strategies, yet this information is largely lacking. This study investigates the homogeneity of a cheese wheel based on the distribution of volatile compounds, microbiota, sodium chloride content, and pH, combined with sensory analyses. The outer zones of the cheese wheel were primarily characterized by the presence of sulfur compounds, esters, pyrazines, ketones, *Streptococcus thermophilus*, high sodium chloride concentration and high pH. In contrast, the inner zones of the cheese wheel were dominated by lactones, carboxylic acids, aldehydes, *Lactobacillus delbrueckii* subsp. *lactis* and *Lacticaseibacillus paracasei*. The presence of alcohols and *Lactobacillus helveticus* was observed throughout the cheese wheel. Furthermore, sensory descriptions were found to match predominantly with the aroma of the volatile compounds identified. The cheese wheel was found to be heterogeneous in all investigated characteristics. Our results indicate that the level of cheese homogeneity should be considered when designing sampling strategies, as these significantly impact the accuracy and reproducibility of analytical outcomes.

## Introduction

Cheese plays an integral role in the gastronomic culture of Europe, offering a diverse range of textures and flavors. Switzerland is a prominent global producer of different cheese varieties, with an annual output of 196,000 tons, of which 76,000 tons are exported. These include among others, hard cheeses, such as Gruyère protected designation of origin (PDO), Emmentaler PDO, and Etivaz PDO. The distinctive sensory attributes of cheese are the result of intricate microbiological, biochemical, and chemical processes occurring during production and maturation.^1,2^ Aroma is characterized by a broad range of specific volatile organic compounds (VOCs) that have a particular impact on human retronasal perception. The balance of these compounds is responsible for the olfactory nuances of each variety.^3^ Given that the first two sensory impressions experienced during tasting are visual appearance and odor, it is plausible that volatile and aroma-active compounds in cheese should be regarded as primordial quality determinants.

The volatile compounds found in cheese arise from the fermentation of milk carbohydrates, as well as from proteolysis and lipolysis during ripening. In addition to contributing to the aroma of cheese, carbohydrates, proteins, and lipids are the basis of cheese texture.^4^ Cheese-making, as a fermented dairy product par excellence, relies nowadays on starter cultures containing lactic acid bacteria (LAB), among which *Lactococcus lactis*, *Lactococcus cremoris*, *Streptococcus thermophilus*, and *Lactobacillus* spp. are the most commonly used.^5,6^ The chemical families that significantly contribute to the overall flavor of cheese include esters, alcohols, aldehydes, ketones, carboxylic acids, sulfur compounds, and lactones. For instance, fruity esters often originate from lactose fermentation, while lipase enzymes catalyze the breakdown of triglycerides into volatile carboxylic acids during lipid degradation in cheese. Proteolytic enzymes derived from specific bacterial cultures facilitate the hydrolysis of proteins into peptides and amino acids, resulting in the formation of volatile sulfur- and nitrogen-containing compounds.^1,7^ Finally, maturation in the cellar allows for the oxidation and reduction of the chemical compounds, leading to the formation of additional, more complex odor molecules. During this stage, the complex and distinctive aromas develop, which are typical for each cheese diary.

The intricate interplay between odorants and sensory receptors in the oral cavity and nasal passages enables the differentiation of cheese varieties. For example, soft cheeses, such as Camembert or Brie, exhibit characteristic mushroom aromas, often fortified by higher concentrations of octen-3-one or octen-3-ol.^8^ Parmesan, renowned for its distinctive umami profile,^9^ contrasts with Emmentaler’s pronounced aroma mediated by its elevated propionic acid content.^10^ Within the same type of cheese, aromas can differ greatly from one terroir to another, or even from one maturing cellar to another, with identical production processes.

In addition to serving as a protective barrier against desiccation, the rind that forms during the ripening process also facilitates specific interactions with the external environment. Microorganisms such as yeasts and molds may develop on the surface of the cheese, contributing to its aroma complexity.^7^ The aroma compounds formed during this process migrate by diffusion through the rind and paste of the cheese. The migration processes are heterogeneous and contribute unevenly to the aroma distribution in the cheese, thus imparting each aged piece a unique character. The technical conditions of production and the bacterial starter cultures used in cheese-making allow, to some extent, for controlling the complexity of the cheese aroma. The manufacturing environment, inherent milk impurities, and whey from previous production all contribute to the manufacturing process and have a major influence on microbial diversity.^11^

A pleasing aroma is a significant factor to be considered by cheese producers, given the paramount importance of consumer satisfaction. Therefore, the chemical, physical, and microbial processes that affect the production of aroma compounds in cheese are the focus of research and routine control measures in the cheese industry and related academic research groups. It is essential to understand the subtle equilibrium of aroma-active compounds present in cheese to obtain a product that accurately reflects the specifications set forth by the producer.

In the case of larger cheese wheels, such as Emmentaler PDO or Gruyère PDO, the aroma profile is hardly homogeneous. As with many other cheeses, they are evaluated by experts using core samples. This procedure is very common in Switzerland for determining the market value of cheeses offered for sale. An unrepresentative evaluation of these cheeses has major financial consequences for the cheesemaker or retailer. From economic, and analytical viewpoints, understanding the inhomogeneity of the sample and implementing a sampling strategy that is aligned with the research question are crucial.

The objective of this study was to gain a deeper understanding of the distribution and homogeneity of volatile aroma compounds as well as the distribution pattern of the cheese microbiota present in Swiss hard cheese. To this end, a specific sampling procedure for large cheese wheels was first developed. Cheese homogeneity was then assessed by analytical mapping of volatile compounds, microbial diversity, physicochemical parameters, and sensory attributes.

## Experimental Section

### Cheese-Making

The details of cheese-making are described in the Supporting Information.

### Sample Preparation for Volatiles, Microbiota and Sensory Analyses

Two cheese wheels aged 9 months were used in this study. Both were produced in a selected cheese dairy in Western Switzerland, known for its quality and reproducibility of production.

**Figure 1:**
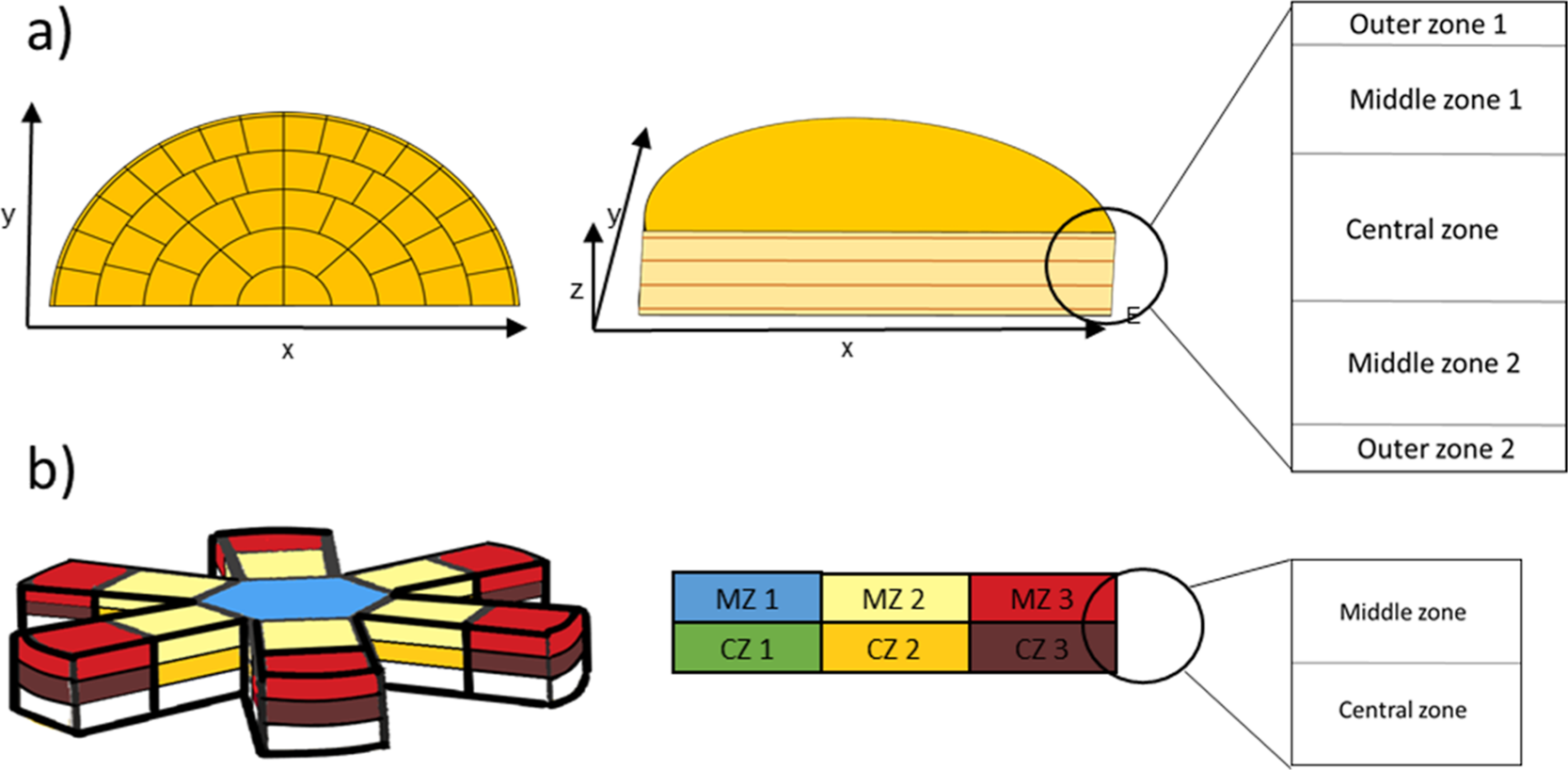
(a) Half cheese sampling on two axes and five layers (outer zone 1 and 2, middle zone 1 and 2, and central zone). Samples used only for aroma compound distribution analyses. (b) Cheese sampling on two axes and two layers (external and internal) without a smear or rind. Samples were used for aroma compound distribution, and microbial and sensory analyses.

The first wheel, produced in 2016 and weighing 32 kg, was divided into two equal parts. One half was cut into 290 samples of approximately 50 g each along three axes (*x*, *y*, *z*). The samples were distributed over five layers, including rind and smear: Outer zone 1 (10 mm), middle zone 1 (25 mm), central zone (30 mm), middle zone 2 (25 mm), and outer zone 2 (10 mm), according to the sampling plan (Figure 1a). For homogenization, each individual sample was frozen in liquid nitrogen and then pulverized in a blender (Robot Coupe Blixer 4 V.V., Pitec, Oberriet, Switzerland). Two grams of each sample were weighed into 20 mL headspace vials (Interchim, Montluçon, France). To prevent oxidation, the vials were purged with argon (Carbagas, Gümligen, Switzerland) for 5 s using two needles to create an “argon stream” inside the vial, which was then sealed with a silicon/Teflon septum (Interchim, Montluçon, France). Samples were then stored in the freezer at −40 °C until analysis, and these preparations were used specifically for volatile analyses.

The second cheese wheel, produced in 2019, was sampled using larger pieces to facilitate various analyses including volatile compounds, microbial counts, physicochemical properties, and sensory evaluations. Sampling was carried out according to the plan illustrated in Figure 1b. Ultimately, 26 representative pieces of cheese without smears and rinds were used for the various analyses. The samples were divided into two parts of two grams for duplicate analysis of the volatile compounds.

### GC–MS Analyses

Samples were randomized using the Excel function RAND, and VOCs were analyzed using vacuum in-tube extraction (V-ITEX) based on the VTT method developed by Fuchsmann et al.^12^ The equipment used consisted of an MPS2 autosampler (Gerstel, Sursee, Switzerland) equipped with an ITEX module (ITEX2, Brechbühler, Switzerland) that included an extraction trap filled with a commercial mixture of Tenax TA/Carbosieve SIII, an Agilent 7890B gas chromatography (GC) system equipped with a programmed temperature vaporizing injector CIS4 (Gerstel, Sursee, Switzerland) and coupled with an Agilent 5977A mass spectrometer (MS) (Agilent Technology, Santa Clara, CA, USA), and Buchi V-300 vacuum pump equipped with an I-300 interface (Büchi, Flawil, Switzerland) operated at 5 mbar.

A piece of 10 × 10 cm cleaned swab Topper 8 (Systagenix, North Yorkshire, United Kingdom) was added to the vial to prevent foaming of the cheese under vacuum. The headspace was extracted for 15 min at 50 °C under reduced pressure (5 mbar) without agitation, keeping the syringe at 100 °C and the trap at 35 °C throughout the extraction process.

Following extraction, the syringe was dried for 17 min and the trap for 5 min under a nitrogen stream (Carbagas, Gümligen, Switzerland) at a flow rate of 220 mL min^–1^. During injection into the CIS4 injector operated in vent mode at 50 mL min^–1^ and 0 kPa for 2 min, the ITEX needle was heated at a rate of 60 °C s^–1^ to 240 °C to desorb the bound volatiles from the sorbent for 2 min under a nitrogen flow of 130 mL min^–1^. The injector was equipped with a glass liner filled with Tenax TA and cooled with liquid nitrogen (Carbagas, Gümligen, Switzerland) at 10 °C. The injector was then heated to 240 °C at a rate of 12 °C s^–1^ to release VOCs into the column. The purge flow to the split vent was set at 300 mL min^–1^ after 5 min. The trap was reconditioned at 300 °C under a nitrogen flow of 130 mL min^–1^ for 15 min. Volatile compounds were separated on a TRB-FFAP fused silica capillary column (100% polyethylenglycol PEG with nitroterephthalic acid, bonded and cross-linked, 60 m × 0.32 mm × 1.0 μm film; Teknokroma, Barcelona, Spain) with helium (Carbagas, Gümligen, Switzerland) as carrier gas at a constant flow of 2.1 mL min^–1^ (37 cm s^–1^). The oven temperature was programmed as follows: 5 min at 40 °C, then ramped to 220 °C at a rate of 5 °C min^–1^ with a final hold time of 14 min, total run time of 60 min. The MS settings were as follows: transfer line at 230 °C, source temperature at 230 °C. The analytes were monitored in full scan mode from 29 to 350 amu with a gain of one and without solvent delay. The autosampler was controlled by Cycle Composer V.1.5.4 (CTC Analytics, Zwingen, Switzerland) and the CIS4 injector with Maestro1 (V.1.4.8.14/3.5; Gerstel, Sursee, Switzerland). The identification of compounds was conducted using the NIST17 library with match factors exceeding 800, in addition to the calculation and comparison of retention indices to the literature found in the NIST database.

### 16S rRNA Amplicon Sequencing

Amplicon libraries were prepared using the unidirectional fusion method (Thermo Fisher Scientific, Waltham, MA, USA). PCR was performed in 50 μL reactions using 4 μL of DNA, 0.1 μM primer NGS_ABCxF27 (5′-CCA TCT CAT CCC TGC GTG TCT CCG ACT CAG |Barcode X| AG AGT TTG ATC MTG GCT CAG–3′), 0.1 mM primer NGS_trP1_355 (5′-CCT CTC TAT GGG CAG TCG GTG ATG CWG CCT CCC GTA GGA GT–3′), relevant region presented in bold, and 45 μL Platinum PCR SuperMix High Fidelity (Thermo Fisher Scientific, Waltham, MA, USA). Amplification was performed as follows: 94 °C for 2 min, followed by 18 cycles of 94 °C for 30 s, 55 °C for 30 s, and 68 °C for 30 s. All amplicons were purified using AMPure XP beads (Beckman Coulter, Brea, CA, USA) with a bead-to-DNA ratio of 1.8. Quality control and quantification of the amplicon library were performed using an Agilent 2100 Bioanalyzer (Agilent Technologies, Santa Clara, CA) and the high sensitivity DNA assay. All amplicons were then prediluted and equimolarly pooled to a final library of 40 pM. Template preparation, chip loading, and sequencing were performed according to the manufacturer’s instructions using the Ion Chef System and the Ion S5 System and an Ion 530 Chip (Thermo Fisher Scientific, Waltham, MA, USA). On average, 320 bp long raw sequences were primer-trimmed and quality-filtered (maxEE = 15, truncQ = 6, maxN = 0, n = 1 × 10^6^, minLen = 100, maxLen = 460) in DADA2.^13^ Amplicon sequence variances were obtained in DADA2 with the parameter POOL = “pseudo” using a previously validated bioinformatic pipeline.^14^ Taxonomic annotation was performed using DAIRYdb v1.2.4^15^ with IDTAXA.^16^ Biostatistical analyses were performed using the PHYLOSEQ package^17^ in R (R Core Team, 2020).

### Absolute Quantification of LAB by qPCR

The assays were conducted in a reaction volume of 12 μL containing the following: 6 μL of Takyon No ROX Probe 2X MasterMix UNG (Eurogentec, Seraing, Belgium), 300 nM of forward primer, 300 nM of reverse primer, and 100 nM of hydrolysis probe. The target genes were *ppC* (*S. thermophilus*), *pheS* (*Lactobacillus delbrueckii* subsp. *lactis*, *Lactobacillus helveticus*), and *recA* (*Lacticaseibacillus paracasei*). The primers and probes utilized for species quantification are listed in Table S1 in the Supporting Information. The qPCR conditions were as follows: 50 °C for 2 min and 95 °C for 3 min, followed by 40 cycles of 95 °C for 3 s and 60 °C for 20 s. All qPCR assays were performed on a Corbett Rotor-Gene 3000 or 6000 (Qiagen, Hilden, Germany).

Serial dilutions of plasmid containing the target sequence or PCR products were included in each run. The DNA concentration was determined using a NanoDrop ND-1000 Spectrophotometer (NanoDrop Technologies, Thermo Fisher Scientific, Waltham, MA, United States). The number of copies corresponding to DNA concentration was estimated using 660 pmol pg^–1^ as the average molecular weight for one nucleotide pair. Data analysis was carried out using Rotor-Gene Q Series Software v2.3.1 (Qiagen, Hilden, Germany), with a threshold of 0.05 for the quantification cycle (Cq) value determination.

### Sensory Analyses

The selection of attributes used for the sensory evaluation was based on established odor associations with odor-active volatiles or groups of volatiles identified in Swiss hard cheese.^18^ The attributes chosen were buttery, milky, sulfury/alliace, cheesy, fruity, flowery, nutty and animalic. An experienced, internally trained cheese panel (*n* = 9), which had undergone several training sessions per year, was additionally trained specifically for this experiment. In the training sessions, the panel tastes different cheeses and describes their attributes. In addition, individual attributes are tasted in both neutral and non-neutral matrices, or trained by nasal smelling. In the special training for this experiment, the selected attributes were trained with nasal perception. To this end, reusable pens (FlavoLogic GmbH, Vaterstetten, Germany) were filled with pure chemical substances representing the chosen flavor attributes (see Table S2 in the Supporting Information). The selected attributes were on an unstructured line scale (10 cm), ranging from “none” at the left end to “strong” at the right end. The panelists were then able to rate the intensity by setting the point on the scale, which was subsequently transformed into numerical values. For sensory evaluation, cheese cubes of approximately 1.5 cm^3^ were cut and stored at 4 °C until the sensory evaluation. In each test session, six cheese samples coded with a random three-digit number were served following a William Latin square design. Noncarbonated water and neutral crackers were provided for neutralization between samples.

All tests were conducted in individual sensory booths at room temperature under daylight conditions. The data was collected using FIZZ software (Version 2.61, Biosystèmes, France) and subsequently analyzed using XLStat software (Version 2019, Addinsoft Inc., USA).

### Physicochemical Analyses

Sodium chloride was quantified by argentometric titration according to ISO 707:2008 in duplicates.^19^ This value in g kg^–1^ was calculated stoichiometrically from the chloride content determined in duplicates. One gram of cheese sample was weighed and transferred to the titration vessel before adding 100 mL of hot Milli-Q (80–90 °C) water. The sample was shaken briefly with a plastic spatula prior to titration, which was performed with an automatic titrator 808 Titrando (Metrohm, Zofingen, Switzerland) and a Titrisol silver nitrate solution at 0.1 mol L^–1^. The pH meter was a Metrohm 605 pH meter (Metrohm, Switzerland) that was previously calibrated with a buffer solution provided by the instrument supplier (pH 4, 7, and 9). The pH was measured between 20 and 25 °C in duplicates by inserting a solid matrix electrode directly into the cheese wheel.

### Multivariate Statistical Analysis

The relationships between the different measured variables were analyzed using multiple factor analysis (MFA).^20^ The analysis values obtained from the second repetition of the cheese wheel were used for the MFA analysis. The panel average was used for the sensory data. Briefly, the data were categorized into three distinct data sets: “Species” (results of the LAB quantification by qPCR), “General” (pH and NaCl concentration), “Volatiles” (results of the GC–MS analyses), and “Sensory” (results of the sensory analyses). The MFA was carried out with the package FactoMineR v.2.8 package using RStudio Pro 2024.04.2 Build 764.pro1^21^ and R software v.4.3.3.^22^

### Terminology of Cheese Zones

Given the 3D configuration of the cheese wheel, the regions were examined in both vertical and horizontal dimensions. For a combined discussion of the results of sampling models (a) and (b) of Figure 1, a consistent methodology is essential. The cheese regions were defined as follows:

- outer zones (0–5 cm under the rind in the *x*- and *y*-directions, and 0–1 cm in the *z*-direction)
- middle zones (5–10 cm in the *x*- and *y*-directions and 2–4 cm in *z*-direction beneath the rind)
- central zone (10–15 cm in the *x*- and *y*-directions and 4–6 cm in the *z*-direction).

Thus, the discussion combines (1) the outer zones of (a) and MZ 3 and CZ 3 of (b), (2) the middle zones of (a) and MZ 2 and CZ 2 of (b), and (3) the central zone of (a) with MZ 1 and CZ 1 of (b).

## Results and Discussion

### General Results: Distribution of Parameters

#### Volatile Compounds

**Figure 2:**
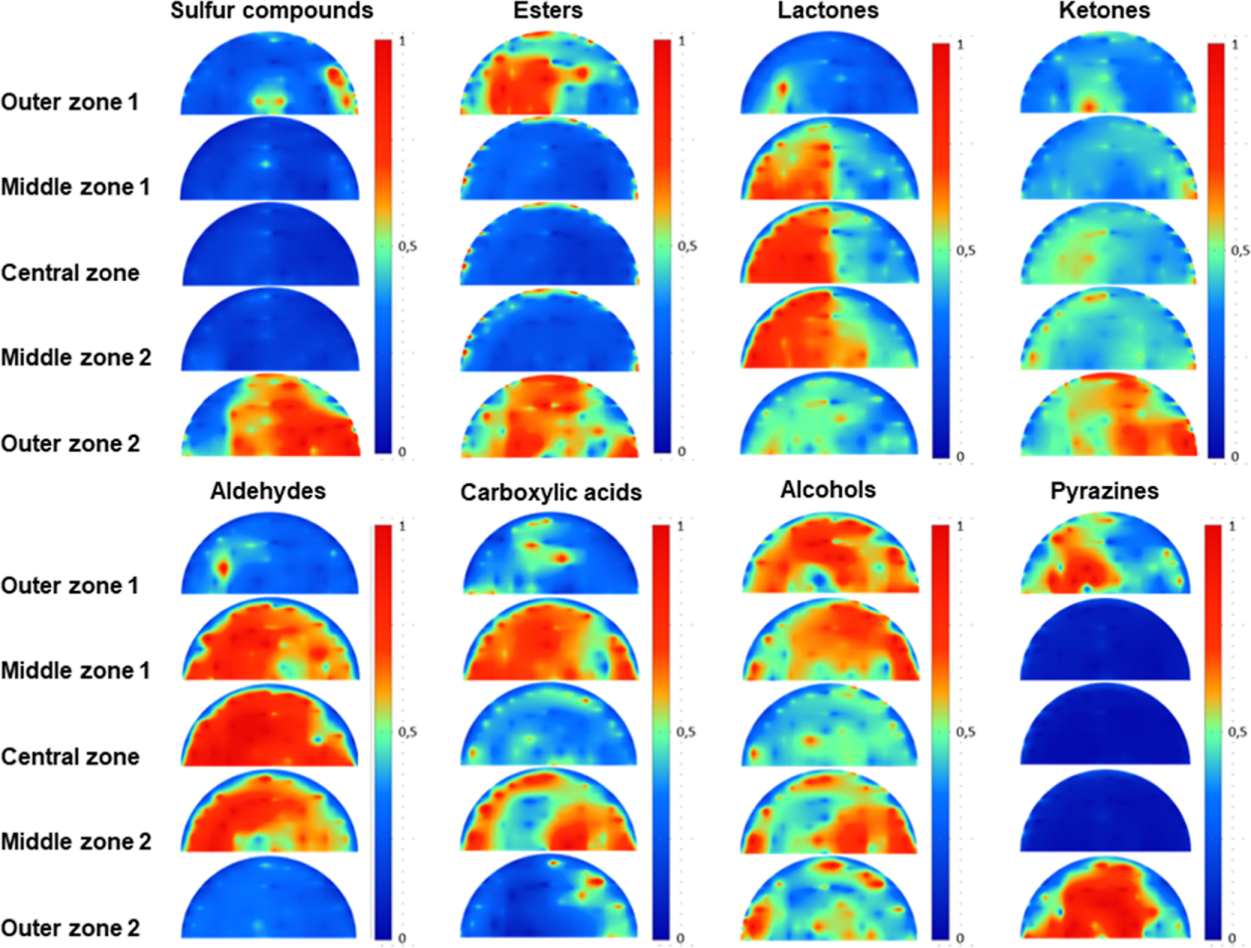
Maps of the different compound groups and their respective intensity distributed over the five cheese zones of the cheese wheel according to sampling strategy (a) (see Figure 1). The colors display the concentration of the compounds, from blue (0, low) to red (1, high).

A selection of 40 volatile aroma compounds representing eight major aroma-active families (alcohols, aldehydes, ketones, lactones, carboxylic acids, pyrazines, esters, and sulfur compounds) is presented in Table 1. The selection of compounds was based on the highest peak area, for which the NIST hit and retention index demonstrated a high degree of identification confidence. To illustrate the variation of the compound groups across the five cheese zones, contour plots were constructed (Figure 2), displaying the normalized intensity from 0 (blue) to 1 (red). The results in Table 1 and Figure 2 were constructed with the first repetition cheese wheel. A second repetition of the mapping results with the second cheese wheel is shown in the Supporting Information in Figure S1. The compounds that were exclusively present in the outer zones, but absent in the inner zones, included sulfur compounds, esters, and pyrazines. By contrast, lactones and aldehydes were exclusively present in the central and middle zones, with the lactones exhibiting localized abundance on a single side. Carboxylic acids were present only in the middle zones but not in the center or outer zones. However, in the repeated analyses, the distribution of carboxylic acids exhibited notable differences (see Figure S1 in the Supporting Information). Alcohols and ketones were observed in all zones.

**Table 1:** Selection of 40 Volatile Organic Compounds Present in the Cheeses with Their Analytical Parameters Retention Time (RT), Retention Index (RI), Literature Retention Index Based on the NIST Library for a Similar Separation Column (Polar Type FFAP), GC Conditions (Ramp Temperature) (Lit. RI), and Qualifier Ions

	compounds	RT (min)	calc. RI	lit. RI	qualifier ion
sulfur compounds	methanthiol	3.44	745	800	48
	dimethyl sulfide (DMS)	3.88	774	777	62
	dimethyl disulfide (DMDS)	10.67	1105	1104	94
	2,4-dithiapentane	16.95	1328	1300	108
	dimethyl trisulfide (DMTS)	19.69	1429	1386	126
	dimethylsulfone	32.48	1978	1890	94
esters	butanoic acid ethyl ester	9.37	1055	1048	116
	hexanoic acid ethyl ester	14.92	1256	1244	115
	octanoic acid ethyl ester	20.41	1457	1431	127
	decanoic acid methyl ester	24.54	1620	1615	155
Lactones	δ-octalactone	33.82	2047	1988	99
	δ-decalactone	38.65	2307	2225	114
	δ-dodecalactone	46.18	2770	2470	99
ketones	butan-2-one	6.24	921	908	72
	pentan-2-one, butane-2,3-dione (diacetyl) coelution	7.99	1002	988/1005	86
	hexan-2-one	8.65	1028	1094	100
	heptan-2-one	13.64	1210	1183	71
	octan-2-one	16.58	1315	1268	71
	3-hydroxybutan-2-one (acetoin)	16.87	1326	1296	88
	nonan-2-one	19.42	1419	1372	142
aldehydes	3-methylbutanal	6.55	936	933	86
	hexanal	10.76	1108	1095	82
	nonanal	19.55	1424	1406	98
acids	acetic acid	21.25	1489	1452	60
	propanoic acid	23.47	1577	1534	74
	2-methylpropanoic acid	24.19	1606	1554	88
	butanoic acid	25.65	1667	1624	73
	3-methylbutanoic acid	26.45	1701	1667	87
	hexanoic acid	30.66	1890	1839	87
	octanoic acid	35.02	2110	2055	115
	nonanoic acid	37.2	2226	2127	129
alcohols	1-butanol	12.3	1163	1179	56
	3-methylbutanol	14.1	1227	1210	70
	1-hexanol	18.24	1375	1361	69
	1-heptanol	20.98	1478	1450	70
pyrazines	2,5-dimethylpyrazine	17.86	1362	1358	108
	2,3,5-trimethylpyrazine	20.05	1443	1394	122
	pyrazine-2-ethyl-3,5-dimethyl	21.05	1481	1435	135
	pyrazine-3,5-diethyl-2-methyl	22.75	1548	1509	149

#### NaCl Concentration and pH

**Figure 3:**
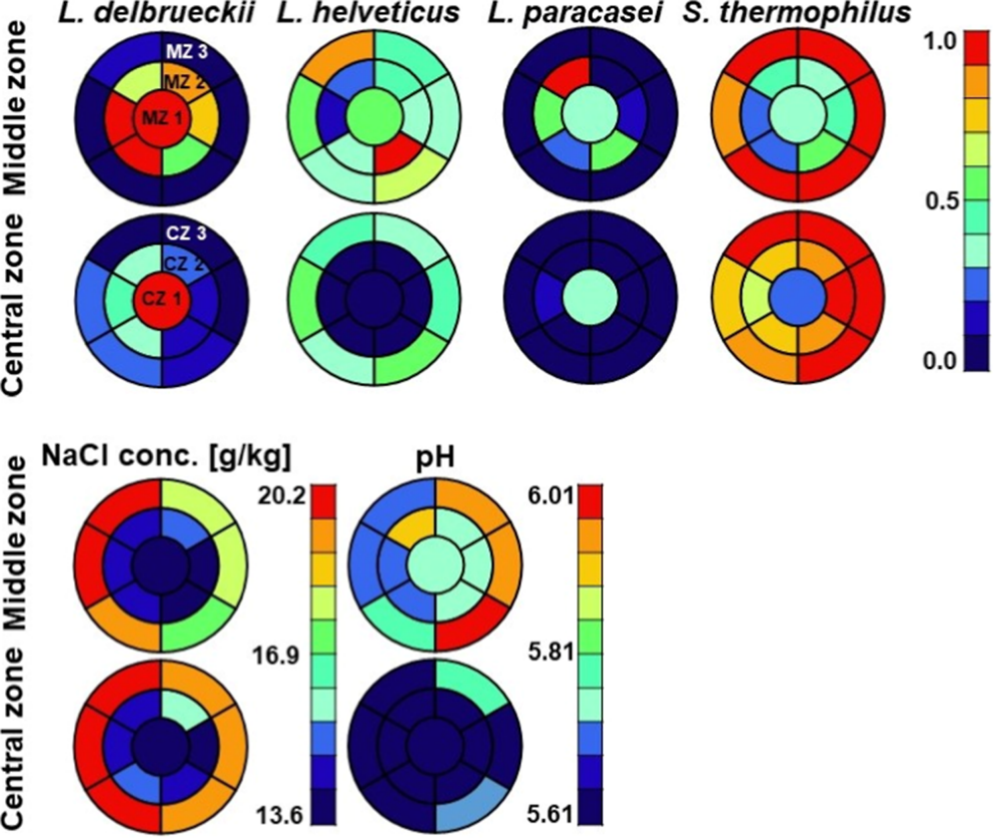
Distribution of *Lactobacillus delbrueckii* subsp. *lactis*, *Lactobacillus helveticus*, *Lacticaseibacillus paracasei* and *Streptococcus thermophilus* determined with 16S rRNA amplicon sequencing and distribution of the NaCl concentration and pH in the middle and central zones of the cheese wheel. The sampling was performed according to strategy (a) (see Figure 1).

The distribution of pH and NaCl concentration are presented in color maps, with blue representing a low concentration and red representing a high concentration. The color gradient transitions from blue to green, yellow, orange, and red, as shown in Figure 3. The highest concentration of NaCl was observed in the outer zones, with a gradual decrease in concentration with increasing cheese depth (Figure 3). The diffusion of NaCl into the cheese is slow,^23^ consequently this can be attributed to the salt bath and repeated application of NaCl solution to the surface of the cheese wheels. The NaCl concentration in the cheese is uneven, particularly on the exterior (ranging from 16.9 to 20.2 g kg^–1^). It can be assumed that the porosity of the cheese rind is not uniform, which may cause the salt to diffuse into the cheese in an irregular manner. The brininess of the initial salt bath, before the rind forms, is also a crucial factor at this stage, as irregular diffusion can occur during this process. Another hypothesis is the presence of moisture on the rind’s surface, which facilitates the diffusion of salt into the cheese. This nonuniform moisture may result from irregular evaporation of the salt solution applied during the brushing of cheeses in the ripening phase.

The pH exhibited a decreasing trend with depth within the cheese, indicating a higher acid concentration in the central zone (Figure 3). This is consistent with the finding of acids in the volatile analysis results shown in Figure 2, although it was not entirely replicate, as shown in Figure S1 of the Supporting Information. The low pH might indicate the influence of lactic acid,^5^ although it should be noted that lactic acid concentration was not measured in this study.

#### Predominant Species

The cheese microbiota was dominated by four common cheese LAB *L. delbrueckii*, *L. helveticus*, *L. paracasei*, and *S. thermophilus* as determined by 16S rRNA amplicon sequencing. The qPCR results were comparable to the 16S rRNA amplicon sequencing results and are presented in the Supporting Information in Figure S2. The predominant species in the outer and middle zones (MZ 3, CZ 3, and CZ 2) was *S. thermophilus* (Figure 3), whereas *L. delbrueckii* was dominant in the central zone. *L. helveticus* was present in the outer and middle zones, although it was absent from the central zone. *L. paracasei* was exclusively present in the middle and central zones. The highest number of total 16S rRNA gene copies was observed in the outer zones (refer to Figures 5 and S3 in the Supporting Information). The distribution of microorganisms within the cheese is not uniform; however, certain trends can be identified. The following sections will address the factors that influence this distribution, including the NaCl concentration, pH, O_2_-content, punctual sources, and species interactions.

A comparison of the logarithmic 16S rRNA gene copies for the four LAB and the total number of copies in the different zones is presented in Figure S3 in the Supporting Information. The data indicated that *S. thermophilus* exhibited the highest 16S rRNA copy number, while *L. paracasei* exhibited the lowest copy number.

#### Sensory Evaluation

**Figure 4:**
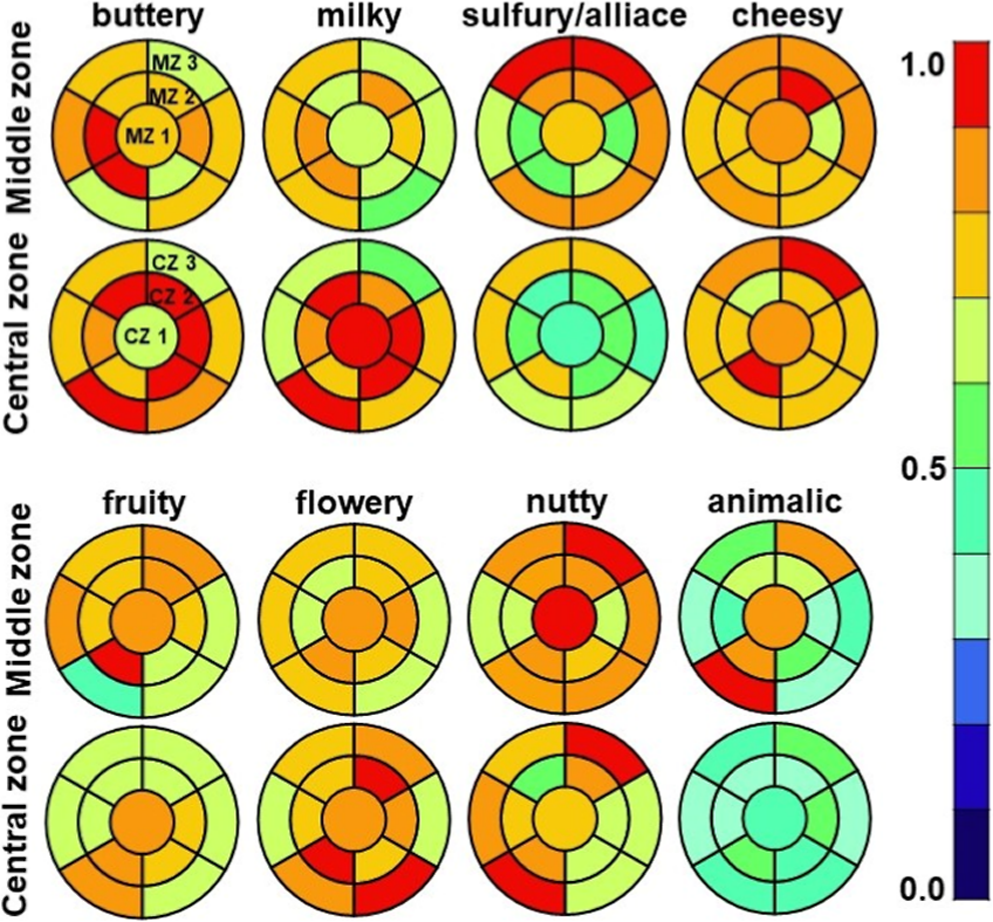
Maps of the sensory attributes of the different zones of the cheese wheel subdivided into major sensory families: buttery, milky, sulfury/alliace, cheesy, fruity, flowery, nutty and animalic. Sampling strategy (a) (see Figure 1) was modified so that the samples of one similar zone were not further subdivided. The panelist ratings were averaged.

Figure 4 depicts the distribution of sensory descriptors (buttery, milky, sulfury/alliaceous, cheesy, fruity, flowery, nutty, and animalic). The outer zones exhibited a pronounced and pervasive general aroma, with pronounced cheesy, sulfury, nutty, and animalic notes. The middle zones were predominantly characterized by flowery, fruity, milky, and buttery notes. The central zone was perceived as milky and nutty.

Table 2 illustrates the common odor threshold ranges for the different compound classes and the sensory descriptors of these compounds. Beyond individual differences, there are also variations in the extent to which panelists perceive various substances. These differences are reflected in the odor threshold, which indicates the lowest concentration of a substance that can be perceived by the human olfactory system. Substances with lower odor thresholds are more likely to be perceived and, consequently, to influence the overall aroma. Among the various chemical compounds, sulfur components, esters (fruity, waxy, fermented), and aldehydes (aldehydic, green) exhibit the lowest odor thresholds and may have the greatest impact on the aroma.

**Table 2:** Odor Thresholds and Sensory Description of Found Compound Classes

compound class	odor threshold range in water (ppb)^24^	sensory description^25^
sulfur compounds	0.0075–6.08	sulfurous, alliaceous
esters	1	fruity, waxy, fermented
lactones	2.5–400	coconut, musk, tropical
ketones	4.4–70,000	buttery, ethereal, fruity, cheesy, earthy
aldehydes	1–4.5	aldehydic, green
acids	240–3000	Acidic, cheesy, fatty, waxy
alcohols	3–2500	fermented, fruity, herbal, green
pyrazines	400–1300	chocolate, nutty, burnt

### Relationships between Variables

#### Volatile Formation: Chemical and Microbial Processes

**Figure 5:**
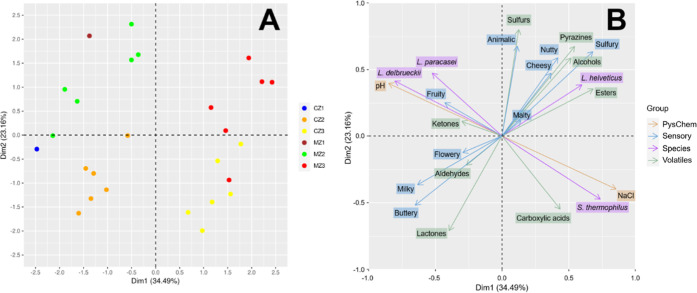
Biplot of the multiple factor analysis (MFA). (A) Plot of the samples described by the combination of variable data sets “Species”, “PhysChem”, “Volatiles”, and “Sensory”; (B) plot of the variables, showing their correlations and contributions to the two first axes of the MFA reduced space.

To assess the relationships between the analyzed variables with no a priori hypothesis on causal relationships, a multiple factor analysis (MFA) was carried out. Figure 5A displays the similarities between samples, described by the variable data sets “Species”, “PysChem”, “Volatiles”, and “Sensory”, and Figure 5B visualizes correlations between the variables and their contributions to the two first axes of the reduced space in the MFA.

The interpretation of the volatile profiles of products derived from fermentation processes is very complex. The formation of these compounds can be attributed to chemical processes (such as oxidation), microbial processes (such as enzymatic activity), or a combination of both.

Volatile sulfur compounds, including methanethiol, dimethyl disulfide, and dimethyl trisulfide, are produced by surface cultures through enzymatic degradation of amino acids, such as methionine and cysteine. Whereas methionine is the primary contributor to the formation of these compounds, cysteine can also undergo enzymatic processes, leading to the production of additional sulfur-containing compounds, contingent on the microbial activities and conditions present during cheese maturation.^26^ The conversion of methionine to methanethiol is a common process among several cheese surface bacteria and LAB, including *S. thermophilus*,*Micrococcus luteus*, and *Arthrobacter* sp.,^27^ and cheese-ripening yeasts such as *Geotrichum*.^28^

Esters can be synthesized in cheese commonly through a variety of processes, including esterification, alcoholysis, acidolysis and transesterification reactions.^29^ These processes are mediated by bacteria, yeasts, and molds during the maturation of cheese.^30^ It has been demonstrated that a coculture of surface bacteria and yeasts is often responsible for ester production, as yeasts provide alcohol substrates for bacterial ester synthesis.^30,31^ Ester synthesis is a common mechanism among various microorganisms, often surface cultures, and is therefore relatively nonspecific. The process has been demonstrated to be relevant in LAB, such as *S. thermophilus* and *L. paracasei*.^29^ In this study, esters were predominantly identified on the cheese surface and within the first 3–5 cm beneath the rind (Figures 2 and S1). *S. thermophilus* was observed in the outer zones MZ 3 and CZ 3, but also in the central zone CZ 2. This finding indicates that *S. thermophilus* alone is not responsible for the ester production and/or that the inner zone conditions are not sufficient for ester production. This evidence suggests that the production of esters by cocultures of surface bacteria and molds are the primary process of ester production.

Pyrazines, represented by 2,5-dimethylpyrazine, 2,3,5-trimethylpyrazine, 2-ethyl-3,5-dimethylpyrazine, and 3,5-diethyl-2-methylpyrazine, are generated during the nonenzymatic Maillard reaction and Strecker degradation during the fermentation and aging processes. Pyrazines originate from amine structures, which are present in proteins, amino acids, and peptides.^32^ Although pyrazines are widely distributed worldwide, the question of whether microbial activities are involved in their formation remains unresolved. Only a few microorganisms appear to be capable of synthesizing pyrazines, with most researchers hypothesizing, that pyrazine formation is in general a nonenzymatic process.^33^ It is unclear which process occurred in this study. However, it is noteworthy that pyrazines were only found in the outer zones of the cheese, suggesting that the Maillard reaction of Strecker degradation is the origin of pyrazines, and that the pyrazines in the inner zones degraded over time. Alternatively, surface cultures, such as *Corynebacterium glutamicum* are capable of pyrazine formation.^34^

The presence of ketones, including butan-2-one, pentan-2-one, butane-2,3-dione (diacetyl), 3-methylbutan-2-one, hexan-2-one, heptan-2-one, octan-2-one, nonan-2-one, and 3-hydroxybutanone (acetoin), at low levels throughout the cheese was accompanied by a higher concentration in the lower cheese layers. These compounds are formed through a variety of metabolic pathways, including lipolysis and subsequent β-oxidation. Prior research has indicated that yeasts and molds on the surface of the cheese are instrumental in catalyzing these processes,^1,35^ and 3-hydroxybutanone was identified as a byproduct of the fermentation process by *S. thermophilus*.^36^ The pathway is as follows: free fatty acids are released during lipolysis and are oxidized to β-ketoacids, which decarboxylate to form ketones. Furthermore, ketones can undergo a reversible reduction to form secondary alcohols under aerobic conditions.^1^ In this context, the production of ketones by *S. thermophilus*, molds, and yeasts in this study is expected to play a significant role, as the highest concentrations of ketones were found in the bottom cheese rind.

The alcohols identified in this study were short-chain alcohols (C2–C8), 1-butanol, 3-methylbutanol, 1-hexanol, and 1-heptanol. These compounds are formed by bacteria, molds, and yeasts in various ways during the refining process, for example, from aldehydes, carboxylic acids, or amino acids via α-keto acids.^6^

Aldehydes, such as 3-methylbutanal, hexanal, and nonanal, are uniformly distributed in the middle zones MZ 2 and CZ 2 (Figures 2 and S1). These compounds can originate from amino acids (through processes such as transamination and subsequent decarboxylation of keto acids) or by Strecker degradation. Both processes were previously observed in the presence of *L. paracasei*.^37^ Aldehydes can be reduced to alcohols or completely oxidized to carboxylic acids, resulting in their typically low concentrations in cheese.^38^ Additionally, *L. delbrueckii* and *L. paracasei* have been identified as producers of aldehydes.^37,39^

The carboxylic acids, including acetic acid, propanoic acid, 2-methylpropanoic acid, butanoic acid, 3-methylbutanoic acid, hexanoic acid, octanoic acid, and nonanoic acid, were predominantly identified in the middle zones (see Figure 2). However, in the second repetition, acids were found sporadically in CZ 3 (see Figure S1). The reason for this inconsistency between the two cheese wheels produced under identical conditions could not be determined in this study. Carboxylic acids can be formed from amino acids or through the oxidation of aldehydes.^6,38^ The microbial pathway originates from amino acids converted to alpha-ketoacids, with subsequent decarboxylation to aldehydes, and further oxidation to carboxylic acids. Additionally, carboxylic acids can act as precursors, for example, for esters, thioesters, cresol, and skatole.^6^*L. helveticus* is the species that produces the highest amounts of carboxylic acids, followed by *S. thermophilus* and *L. delbrueckii*, with the latter producing only low amounts of carboxylic acids.^40^

The distribution of lactones, including δ-octalactone, δ-decalactone and δ-dodecalactone, was uneven across middle zones 1 and 2, MZ 2, CZ 3, CZ 2, and CZ 1 (refer to Figures 2 and S1). This uneven distribution contrasts with that observed for other compound classes, suggesting that lactone production is a specific and potentially slow process, likely originating from localized sources, such as a specific culture within the cheese. Lactones are formed through the intramolecular esterification of hydroxy free fatty acids.^1^ The hydroxylation of the free fatty acids may result from normal fatty acid catabolism or in the presence of enzymes such as lipoxygenases or hydratases.^41^

*L. delbrueckii* is the predominant species in the central zone and is associated mainly with the production of carboxylic acids and ketones (see Figure 5). The species is known to enhance proteolysis, contributing to a less sweet taste and a more elastic texture of the cheese.^39,42^

#### Microbiota: Species Interactions and Influence of Other Parameters

A review of the distribution of *S. thermophilus* and *L. delbrueckii* suggests the potential negative influence of these two species on one another (Figure 3). *S. thermophilus* is present in all regions where *L. delbrueckii* is absent, and vice versa. Strains of *S. thermophilus* and *L. delbrueckii* are common hard cheese starter cultures and are known to interact with each other in a variety of ways, including competition and amensalism, which are often facilitated by bacteriocin production. Alternatively, these strains may engage in mutualism through protocooperation as is the case in Joghurt.^43,44^ However, the results of this study exhibited *S. thermophilus* greater tolerance to neutral pH and oxygen levels, leading to higher counts in areas with these characteristics (Figure 3). Conversely, *L. delbrueckii* is known to display a preference for lower pH and reduced oxygen availability.^44^

We observed a pH gradient from outside (high) to inside (low), as shown in Figure 3, which could be of microbial origin. LAB are known to contribute to the acidification of the cheese during milk fermentation due to the production of lactic acid.^5^ A higher pH on the outside of the cheese and a pH gradient from outside to inside have been observed in mold-ripened cheeses, such as Camembert.^45^ In such cases, the lactic acid present on the surface of the cheese is metabolized by surface cultures, resulting in the production of water and CO_2_ and/or ammonia, which increases the pH of the exterior of the cheese. It is reasonable to hypothesize that the surface cultures also influenced the pH in this study.

The use of NaCl to inhibit the growth of microorganisms that can impair food quality is a well-established technique in food fermentation. The impact of salt on cell viability and autolysis has been demonstrated to be more pronounced than that of cooking temperature.^46^*S. thermophilus* is generally highly resistant, and studies have demonstrated that salt and cooking temperatures of 40–50 °C had no effect on this species. By contrast, *L. helveticus* is significantly influenced by salt, and, to a lesser extent, by cooking temperature. In cheeses with high salt concentrations, this species exhibited reduced viability, autolysis, and proteolysis.^46^ In addition to the effects mentioned above, this could explain why *S. thermophilus* was almost exclusively found in the outer zones.

#### Sensory Profile: Influence of Volatiles

As the sensory analyses were conducted solely on the cheese wheel from the second repetition, the distribution of volatile compounds from the same repetition (see Figure S1 in Supporting Information) was utilized for interpretation. In general, the areas close to the cheese rind exhibited the highest concentrations of aroma-active compounds, which contributed to a pronounced overall flavor profile (Figure 5). These observations can be attributed to the influence of external parameters, including the cultivation of the cheese surface, aerobic conditions, and rubbing with a NaCl solution. Guillen and Abascal also observed a greater degree of variability in volatile compounds within the outer zones of cheeses.^47^

Buttery and milky notes are generally attributed to ketones, lactones, or aldehydes.^6,37^ These aroma notes were observed in the outer, middle, and central zones, but they were more concentrated in the inner zones. In this study, the buttery note can therefore, be linked to the presence of aldehydes, ketones, and partly, lactones (see Figure 2).

The sulfur compounds are responsible for imparting sulfury and/or alliaceous notes.^48^ The flavor description aligns with the observed presence of sulfur compounds, as both were found in the outer zones of the cheese, with decreasing concentrations in the inner zones.

A cheesy note was predominantly observed in the outer and middle zones. This sensory attribute may be associated with acids, ketones, and sulfur-containing acids.^48,49^ Carboxylic acids and sulfur compounds were identified in the outer zones, whereas ketones were present in the outer and middle zones (see Figures S1 and 2).

The introduction of fruity notes is typically attributed to the presence of esters. The highest concentrations of esters were observed in the outer zones. It is noteworthy that the fruity note typically associated with ester compounds was most pronounced in the middle zone of the cheese. This discrepancy between the sensory and analytical results may be attributed to the overall stronger aroma of the outer cheese zones, which may have masked the fruity notes. Furthermore, the presence of ketones or alcohols can evoke fruity notes.^25^

The outer and middle zones were described as having a flowery aroma. Researchers have previously demonstrated that ketones, aldehydes, and alcohols have the potential to impart floral notes.^50^ In this study, aldehydes and ketones were identified in the outer and middle zones, whereas alcohols were exclusively present in the outer zones.

The nutty flavor is primarily associated with pyrazines.^25^ Regions that were described as nutty in the sensory analyses were the outer and central zones. Pyrazines were exclusively present in the outer zones. Pyrazines, which produce intense hazelnut aromas, have been identified as a primary contributor to the aroma of the first millimeters beneath the cheese rind.^32^ However, it remains unclear whether the nutty flavor observed in the central zone was the result of a single compound or a combination of compounds. In other studies, nutty flavor has been linked to various compound classes, indicating a relatively nonspecific attribution. These include ketones, lactones, esters, alcohols, aldehydes, pyrazines, sulfur compounds, carbonyl compounds, fatty acids, amino acids, and salts.^51^

The outer zones of the cheese were described as exhibiting an animalic flavor, which is known to correlate highly with the presence of carboxylic acids.^52^ Indeed, the predominant presence of carboxylic acids in the repeated measurements (see Figure S1 in Supporting Information) was observed in the outer zones of the cheese, with minimal detection in the inner zone.

The results of this study illustrate a notable variation in the distribution of volatile compounds, microbiota, pH, NaCl concentration, and sensory description across the cheese samples. This demonstrates the necessity of ensuring the proper homogenization of the cheese samples for the attainment of reliable analytical results. The findings of this study unequivocally illustrate that a whole cheese cannot be regarded as a homogeneous entity. A multitude of factors contribute to the formation and release of aromas, which exhibit notable variation across different zones of the cheese. These findings are of great consequence for the optimization of sampling strategies for the analysis of cheese. Furthermore, they contribute to a deeper comprehension of the interactions between microorganisms and the physical parameters of cheese, which ultimately influence the formation of volatile compounds, particularly those responsible for the perceived aroma. To investigate specific parameters, it is essential to determine whether the objective is to identify differences throughout the cheese or to focus on the cheese in its totality. Consequently, the sampling strategy is contingent on the research question. In the context of sensory evaluation, it is of paramount importance for the cheese producer to obtain a representative evaluation of the cheese, which cannot be fully achieved using a single piece of cheese; rather, representative samples should be taken across the entire cheese. It is recommended that three-to-five pieces be taken from different layers of the cheese, including the outer zone, the central zone, and the middle zone.

## Supporting Information Available

Details on Cheese making. qPCR primers and probes in Table S1. List of compounds used for sensory panel training in Table S2. Distribution of volatile compounds of the second repetition in Figure S1. qPCR results in Figure S2. 16S rRNA copies and total amount in the different zones in Figure S3 (PDF)
