# Supplementary material for: Analytical Mapping of Swiss Hard Cheese to Highlight the Distribution of Volatile Compounds, Aroma, and Microbiota
Source: J Agric Food Chem. 2025 Mar 11;73(12):7383–92. doi: 10.1021/acs.jafc.4c10980 (PMC11951147; doi:10.1021/acs.jafc.4c10980)
Supplement: Supplementary file 1 — jf4c10980_si_001.pdf [file jf4c10980_si_001.pdf]

Supporting information to "Analytical mapping of swiss hard cheese to highlight the distribution of volatile compounds, aroma, and microbiota"

Lucie K. Tintrop[a*], Marco Meola[b], Mireille T. Stern[a], Monika Haueter[c], Noam Shani[a], Hélène Berthoud[a], Barbara Guggenbühl Gasser[a], Pascal Fuchsmann[a]

[a]Agroscope, Schwarzenburgstrasse 161, 3003 Bern, Switzerland
[b]DATABIOMIX, Zürcherstrasse 39D, 8952 Schlieren, Switzerland
[c]Swissmedic, Hallerstrasse 7, 3012 Bern, Switzerland

*Corresponding author
Lucie K. Tintrop, Agroscope, Schwarzenburgstrasse 161, 3003 Bern, Switzerland, Phone: +41 58 465 79 34, E-Mail: lucie.tintrop@agroscope.admin.ch

Cheese making

The cheeses were made in the same cheese factory from unpasteurized and non-thermized raw milk. All manufacturing parameters follow strict specifications. The cheeses produced were about 10 cm high, about 60 cm in diameter, and weighed 32 kg.

Half of the raw milk used to make the cheese came from the evening milking which had been stored at 15°C and mixed with the milk from the morning milking. The mixture was standardised by adding skimmed milk in order to obtain a fat content 0.2 % higher than the protein content. The milk was then matured at 31°C for a period of 20 min. After maturation, a cheese culture mixture was added with natural calf rennet to the milk. This mixture contained (i) 1/3 of an Agroscope starter culture bulked on sterile milk and composed of strains of the thermophilic lactic acid bacteria *S. thermophilus* and *L. delbrueckii* ssp. *lactis* and (ii) 2/3 of a natural whey culture obtained by back-slopping from the previous day's production, incubated at 38°C for 20 h, and containing strains of *L. helveticus*. Cultures from the previous day's whey contain a wide variety of species and are specific to each cheese dairy. Coagulation of the milk took place over a period of 40 min at 31°C. The curd was cut with a curd slicer into grains the size of wheat grain. The temperature was then raised to 57°C for a period of 40 min to remove some of the whey from the grains. The grains were stirred for 10 min before being pumped and transferred to moulds. The moulds were put under pressure for 24 h to obtain a good cohesion of the curd grains.

The cheeses were immersed in a brine bath of 21°C for 24 h and then transferred to a cellar at 14°C and 92 °RH (relative humidity) for maturing. The cheeses were turned over and rubbed with a salted solution (1-5 times a week) regularly throughout the maturing period, which lasted 9 months.

qPCR primers and probes

Table S1 PCR primers and probes used for species quantification.

Species	Primer name	Primer sequence (5'-3')	Target gene
Streptococcus thermophilus	Stherm fw	TGCACAGTTGGCAGAGAGTGA	phosphoenolpyruvate carboxylase (*ppC*)
	Stherm rev	TGCCACTCATCCAAAATGATG	
	Stherm MGB FAM	AAGTTCGTAGCGTCTTT	
Lactobacillus delbrueckii	Lbdel pheS F2	GAA AAG CGG CGG GAA GTC	phenylalanyl-tRNA synthase alpha subunit (*pheS*)
	Lbdel pheS R	CAA TGG CCT TTT CGA TCA AGT T	
	Lbdel pheS FAM	TCAACGAACTCCGCGACCTCTTTAATG A	
Lactobacillus helveticus	Lbhelv pheS F3	AGGTTCAAAGCATCCAATCAATATT	phenylalanyl-tRNA synthase alpha subunit (*pheS*)
	Lbhelv pheS Rmgb	TTTCGGGACCTTGCACTACTTTA	

	Lbhelv pheS MGB FAM	CTACTTCATCGGTATGGGTT	
Lacticaseibacillus paracasei	Lbcasei recAF	TTATGCGAATGGGTGCTAAGG	recombinase A (*recA*)
	Lbcasei recAR	CCAACACCAAGTGCATCATCA	
	Lbcasei recAFAM	CGTTTCCGTTGTCTCTAGCGGCTCACT	

Compounds used for sensory panel training

Table S2 Compounds and associated sensory descriptor used for panel training.

Sensory descriptor	Chemical compounds
Buttery	Pentane-2,3-dione
Animal	3-Ethylphenol
Cheesy/rancid	Butanoic acid
Fruity	Ethyl butanoate
Cooked/roasted	Furfural
Sulfury/alliaceous	Dimethyl trisulfide
Floral	2-Phenylethanol
Malty	3-Methylbutan-1-ol
Mushroom/underwood	Oct-1-en-3-one

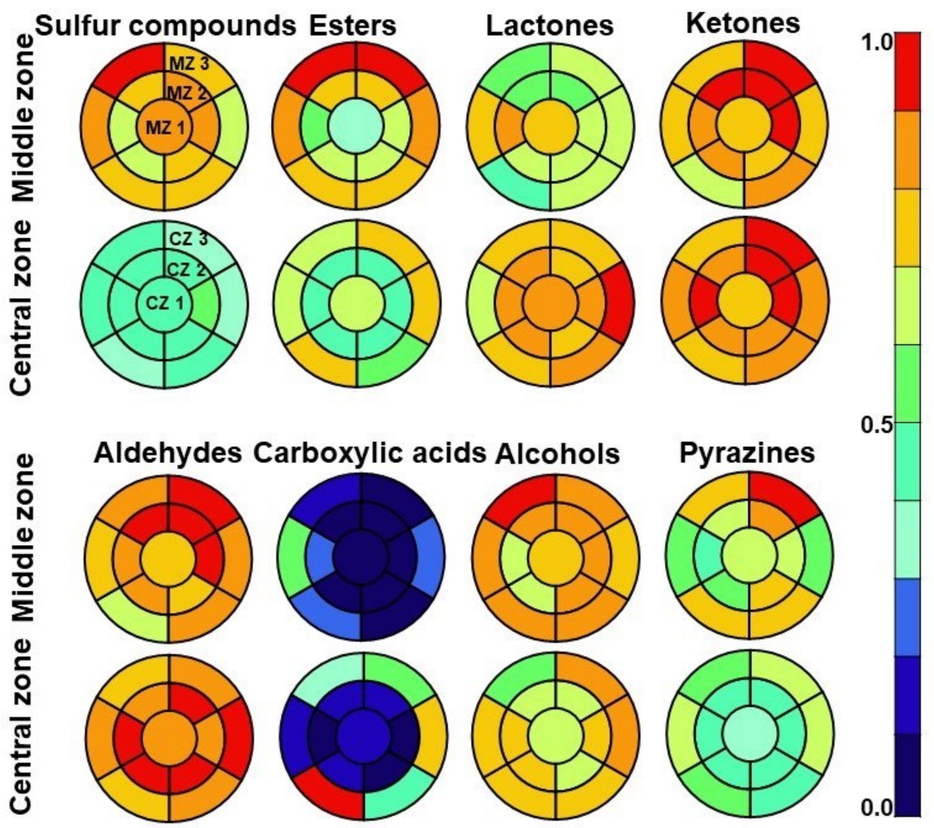

Figure S1 Distribution of volatile compounds in the middle and central zones from the cheese wheel of the second repetition. The used sampling strategy is described in Figure 1 (a).

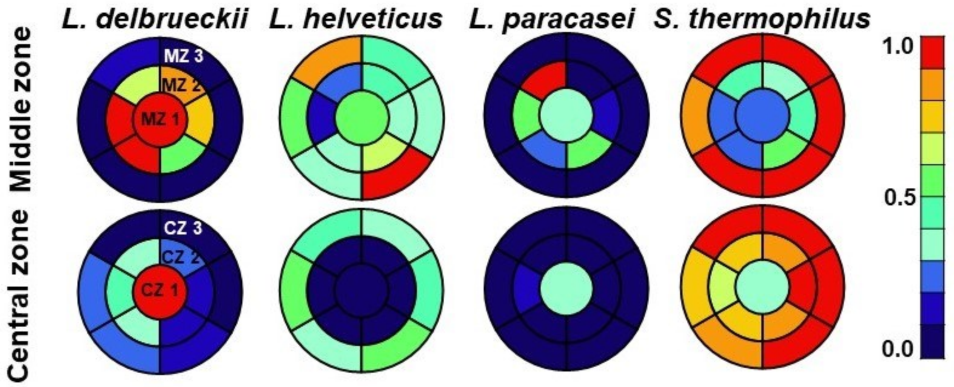

Figure S2 Distribution of *Lactobacillus delbrueckii*, *Lactobacillus helveticus*, *Lacticaseibacillus paracasei* and *Streptococcus thermophilus* determined with qPCR. The sampling was performed according to strategy (a) (see Figure 1).

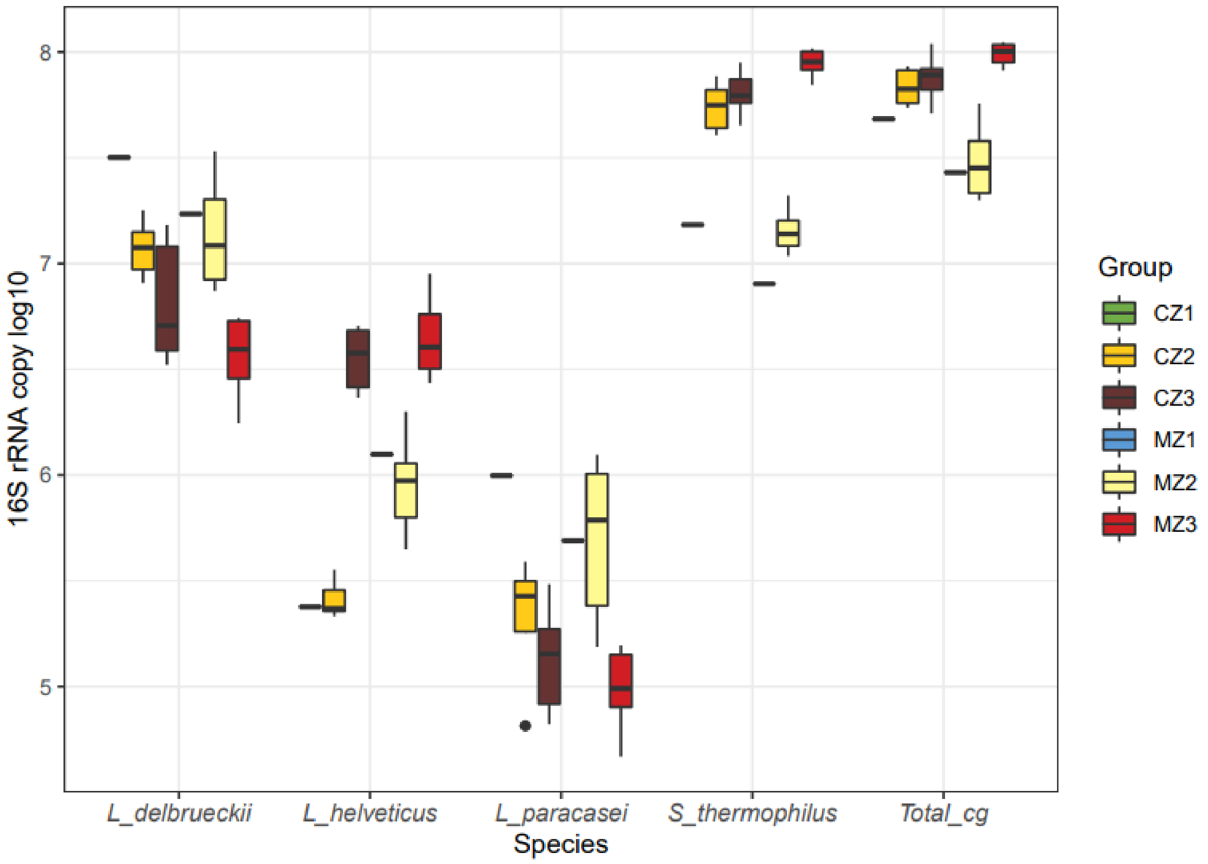

Figure S3 Logarithmic 16S rRNA copies of the four different bacteria and the total amount in the different zones.
